# An Eulogium on William P. Dewees, M.D. Delivered before the Medical Students of the University of Pennsylvania

**Published:** 1842-11-26

**Authors:** 


### MEDICAL EXAMINER.

NEW SERIES.

No. 48.] PHILADELPHIA, NOVEMBER 26, 1842. [Vol. I.

### BIBLIOGRAPHICAL NOTICES.

*An Eulogium on William P. Dewees, M. D. Delivered before the Medical Students of the University of Pennsylvania.* November 5, 1842. By Hugh L. Hodge, M. D. Professor of Obstetrics, &c. &c. Philadelphia: 8vo. pp. 58.

Dr. Dewees was of Swedish descent, his family being among the early settlers of the Delaware Bay and River, before the time of William Penn. He was born May 5th, 1768, at Pottsgrove. His father died whilst he was quite young, and left him with little property and a slender education. "He is represented by those best calculated to judge, as docile, industrious, very affectionate and amiable," (p. 8.) He commenced the study of medicine early under Dr. Phyle, a practising apothecary, with whom he remained two or three years, and then entered the office of Dr. William Smith. In the years 1787, 1788, and 1789, he attended the lectures of the University of Pennsylvania, the professors at that time being Drs. John Morgan, William Shippen, Adam Kuhn, and Benjamin Rush. At that period it was almost an universal custom to practise without a diploma; and in the summer of 1789 Dr. Dewees commenced his professional life without being a graduate, at Abington, fourteen miles north of Philadelphia.

"He was then twenty-one years of age, about the medium height, well proportioned, of a florid complexion, dark hair, rather slender make, and remarkably youthful in his appearance, so that great objections were frequently made to employing a physician apparently so very young."

The first yellow fever which desolated our city thinned the ranks of the profession, and a fine field of practice was offered, and Dr. Dewees removed to this city in 1793.

"At that period, the science of obstetrics was hardly known in America. The physicians who occasionally engaged in its practice, had received no instruction, with the exception of a few who, having visited Europe, brought home a general knowledge of the subject, but who, from the prejudices existing against the employment of male practitioners, had few opportunities and fewer inducements to perfect their knowledge. Hence, midwifery existed almost universally as an art: the aged and imbecile nurse was almost universally preferred to the physician. Women were generally the practitioners of midwifery, as few imagined any particular instruction necessary for an attendance on labour; at least any beyond that derivable from prolonged experience. Our science, however, gentlemen, is too essentially connected with the lives and happiness of individuals and families, to remain, for a long time, in such obscurity, when knowledge and science on other subjects were elevating the character and developing the resources of the community. As the arts and luxuries of life increased, the dangers and difficulties of the parturient process increased also. Experience lamentably demonstrated that the attentions of the nurse, however experienced, were unavailing; yea, that the officious interference of ignorant practitioners in a process so wonderful and so abstruse as that of parturition, was too often productive of the most fatal consequences to the child and its mother, thus destroying the comfort and happiness of families. In such extremities, all notions of false delicacy are thrown to the winds—the cry for help arising from the emergencies of the case is imperative; but, alas, who was prepared to respond to the cry? who to render the necessary assistance? The physician who, on such emergencies, was called, was unprepared to afford relief; his former studies had been imperfect; his experience in midwifery trifling; his observation of severe cases very limited; and you may imagine the embarrassing and horrible condition in which such a practitioner must be placed, when a human being, and that a female, in agony, supplicating for relief—when to him every eye was turned—when on him rested every hope of a despairing husband or a broken-hearted mother, and he felt conscious that he ought to be able, but still could not afford the proper assistance. Such was the condition of our community some fifty years ago—such, we are sorry to affirm, is the state of many communities, in various portions of our country, at the present day—where often, very often, the cry for help bursts from the agonized bosom, and there is no suitable response from the instructed obstetrician.

What greater incitement could be offered to a young medical man, conscious of power, but sensible of his deficiencies, than such a state of things? What more extensive field of usefulness could be presented to a conscientious and philanthropic youth, burning with desire to benefit his race, than to labour for the preservation of mothers and their children during the eventful and agonizing moments of parturition?

The opportunity, thus Providentially occurring, was embraced by the subject of our address. He felt and realized his own deficiencies, but was determined to overcome them; and, in after life, reviewing the difficulties of his situation at that time, the obstacles in his progress, arising from the ignorance and prejudices of the community, the imperfect state of obstetric science, and his own deficient education, he might well adopt the language of the celebrated conqueror of antiquity—"*Veni, Vidi, Vici!*"

To attain the victory—to prepare himself for the elevated station to which he aspired—could only be effected by rendering himself equal to the emergency. He reviewed his observations, made during four years at Abington, at the bedside of his patients,—he compared these results with the experience of others: he went still further; he commenced again an examination of the foundations of his science, the fundamental principles of obstetrics; and on these he built his stable superstructure, which has, and will last, to his own credit, and to the reputation of our school, our city, and our country. He made himself familiar with the then modern authorities—the Osbornes and Denmans of England, the Levrèts and Baudelocques of France; and hence derived accurate notions of the science and practice of midwifery.

His investigations, when compared with the results of his own experience, excited a partiality for French in preference to English obstetrics. He chose Baudelocque for his teacher; and often declared that he was indebted to this most distinguished French obstetrician, for all that he himself knew of midwifery. The disciple was worthy of his master.

Thus armed for the conflict with the ignorance and prejudices of the community—with the irregular, the uneducated, or the imperfectly educated practitioners of the art, he was ready for the emergencies that might occur. Such emergencies were not unfrequent; for, unfortunately, difficult cases of delivery were at that period the result, not only of natural causes, but very frequently of the bad and officious practice of ignorant pretenders to the art, who made that labour difficult or laborious, which, without their interference, would have been natural and comparatively easy. On such occasions Dewees was often consulted. He had succeeded in rendering himself a favorite with the midwives, by the kindness of his manner, while he maintained the supremacy of his profession in immediately taking the cases exclusively into his own hands, allowing them no further agency than as mere nurses or assistants. Still he did not run counter to their prejudices, or apparently act in opposition to their interests; hence he was frequently sent for, and a large portion of operative midwifery fell into his hands. For him, this was in every way advantageous; his theoretical knowledge became practical—his dexterity in operating, as well as his tact in the difficult art of diagnosis, was perfected; his reputation was diffused through the community, and his practice, of course, became more extensive and profitable. In a short period, therefore, after his establishment in Philadelphia, under the conjoint influences of the causes mentioned, but especially by his own real worth and decision of character, his success was complete, and he felt that he might safely enlarge his responsibilities and assume new duties, while he added to his comforts and happiness."

About this time he married the daughter of Dr. Rogers, of New England, who "was young, lively, animated, and very beautiful." Soon after her marriage she fell a victim to an acute disease. About the year 1800, Dr. Dewees commenced his career as a teacher, and was the first regular lecturer on obstetrics in our country.

"He again determined to seek the advantages and pleasures of domestic life, and in the year 1802 became united to his second wife, Miss Mary Lorrain, daughter of John Lorrain, long a respectable merchant in Philadelphia. In this connection, he was greatly blessed: Mrs. Dewees was preserved in health and strength as the partner of his prosperity and adversity, enjoying with him the innumerable favours which Providence, in the course of a long life, had abundantly bestowed, and sharing with him those painful reverses that occurred in the latter periods of his life. By this marriage, Dr. Dewees became the father of eight children—three daughters and five sons—most of whom survive him."

On the 11th of April, 1810, the Board of Trustees of the University of Pennsylvania erected a separate Professorship of Obstetrics, the chair having heretofore been merged in that of Anatomy and Surgery, held by Dr. Shippen, and being, in fact, merely nominal.

"The struggle for the new chair in the University was very warm, and the claims of opposing candidates, and the influence of their respective friends, rendered the event doubtful. The strong claims of Dr. Dewees, his talents, his industry, his attainments, his dexterity, boldness, decision, and judgment as a practitioner, his great success in the practice of his art, and as a teacher of its principles, his popularity, supported by the strongest testimonials from many of the distinguished men in the profession, including Rush and Physick, were met by analogous claims of opposing candidates, Dr. James, and Dr. Chapman.

On the 29th June, 1810, the decision was made by the election of Dr. Thomas C. James to the new Professorship, the first in this country.* This disappointment to the long cherished hopes and expectations of Dr. Dewees was certainly great, but involved no loss of character, as the most ample testimony was borne as to his qualifications and character, and the public confidence in his skill was entirely unabated. It could only be said that his influence with the Board of Trustees proved to be weaker than that of his rivals."

His devotion to the arduous duties of his profession, " loss of sleep, irregular hours, laborious occupation, and continual mental and moral excitement," impaired his health, and he was threatened with a pulmonary affection, and was induced, by the tempting offers of successful pecuniary investments, to remove to Phillipsburg, Pa. His health was restored, but his property was lost, and he returned, in 1817, to the city, with a large family, and very needy.

" His immediate wants being supplied by the kindness of professional friends, he resumed his private course of instruction to medical students on midwifery, and the practice of his profession. He soon became connected with Drs. Chapman and Horner in conducting that system of private instruction, which for years has been, most advantageously, conducted in our city, under the denomination of the Medical Institute of Philadelphia, founded originally by Dr. Chapman, about the year 1817, and which yet flourishes under an enlarged and efficient organization. To its success Dr. Dewees greatly contributed, from the period mentioned until 1832, when age, and other pressing circumstances, induced him to resign."

It was about this time that he commenced recording his experience, the fruits of which were the publication of several volumes, whose admirable practical worth will be acknowledged by the American practitioner. No American physician was better known or more appreciated abroad than Dewees, and that chiefly through his writings. On the 15th Nov., 1825, he was appointed adjunct professor to Dr. James, in the University of Pennsylvania, whose declining health demanded assistance.

*This appointment was made with the understanding that Dr. Chapman should assist the Professor in his lectures,—receive half of the emoluments, and, on the occurrence of a vacancy in the University, be appointed to fill the vacant chair. All these conditions were fulfilled—and, on the death of Dr. B. Rush, in 1813, when Dr. Benj. S. Barton was appointed his successor, Dr. Chapman received the unanimous vote of the Trustees, as Professor of Meteria Medica and Pharmacy.

"Dr. Dewees, on his entrance into the University, was fifty-seven years of age, in full possession of his mental and corporeal faculties. His figure had spread considerably,—so that he could be termed portly,—while he maintained a comparatively youthful appearance, from his florid complexion and dark hair, still without the silvery gloss of age. The duties of the professorship gradually devolved more and more upon him as Dr. James declined in health, and were discharged in a manner very acceptable to the students. Of course, there was no great display of eloquence or erudition in his lectures, but he was always clear, decided, precise, and minute in his directions; speaking in rather a conversational style, with the promptitude and confidence of a man who had formed his own opinions by his own observations, and illustrating all that he taught by a rich fund of cases and anecdotes, drawn, in a great measure, from what he had himself witnessed. Such a teacher could not be otherwise than interesting, and, from the whole character of his mind, with its endowments, natural and acquired, you may readily conclude, he must have been exceedingly valuable. His popularity was great, and his usefulness became thus greatly extended; his pupils distributing his fame, as well as his valuable instructions, through the extent of our country."

In February, 1834, a slight accident changed this career of prosperity. The necessary confinement induced apoplexy, but the alarming symptoms soon yielded under the management of Drs. Chapman and Hays. In the autumn of the same year, Dr. James resigned the Chair of Obstetrics, and Dr. Dewees was unanimously appointed. The duties of the ensuing winter were, however, too severe, and on the opening of the course of 1835–'36, it was evident that he would be unable to continue the lectures. We well remember the reluctance of the class to acknowledge this melancholy fact, and how they all clung to the hope that something might occur which would prolong his connection with the school. All was, however, in vain; and when the inevitable resignation was announced, never was genuine regret more sincerely felt, or more warmly expressed. A subscription was immediately set on foot for the purchase of a magnificent vase; and on the 25th of November, 1835, the beloved professor took leave of a class, the older members of which were parting with a friend and preceptor, who was accustomed to pour forth his valued lore, with a happy fluency and apt illustration, which sent away his hearers charmed and instructed by his talents and acquirements, and won by his amiable and affectionate disposition. The parting scene was too trying to the shattered system of the illustrious sufferer, and his venerable colleague and well-tried friend, Professor Chapman, bade farewell in his name. He embarked immediately for Havana, and from thence went to Mobile, where he resided until March, 1840, when he returned to this city, and died, May 22, 1841.

"In the practice of obstetrics, the boldness and decision of Dr. Dewees was of vast importance. There was no rashness in his efforts, because he took the essential precaution of studying the science of midwifery, before he ventured on the difficult points of practice. He imbibed the best principles from the best teacher—Baudelocque of France,—and thus furnished, he had no want of confidence in himself, or his art, in any emergency. He never drew back and allowed his fellow beings to perish, when the means of relief were at command, for fear of danger or responsibility. All that could be done was done, and well done; and I have the authority of my now venerable colleague, Dr. Chapman, the fellow labourer of his friend, Dr. Dewees, for declaring, that no man was a better or more successful obstetric operator than Dr. Dewees, especially in the use of the forceps. The consequence has been, that not only did our predecessor accomplish a vast amount of good himself by means of operative midwifery, but he has, by his example and his instructions, vindicated this branch of the profession from the reproaches of the timid or ignorant practitioners, who were so terrified by the horrible consequences of mismanaged labours, as to dread the employment of artificial measures, even in cases of acknowledged difficulty. The daughters of America, in this respect owe, and will always owe, an immense debt of gratitude to their true friend—Dr. William P. Dewees.

"But, gentlemen, you will hereafter find, that something more than mere talent and force of character is demanded to insure success as practitioners of medicine, particularly for those whose attention is devoted to the sufferings and diseases of the more delicate portion of the human family. The qualities of the heart must be superadded to talent and wisdom. In this respect, Dr. Dewees was not deficient; although not remarkable for polish of manner or refinement of character, he had warm affections, became deeply interested in his patients, sympathized with their sufferings, and, by the kindness of his manner and the earnestness of his attentions, impressed them with the belief, that all the energies of his character, and all the resources of his profession were devoted to their relief. He was peculiarly happy in his conversation with his patients: having a cheerful, pleasant disposition, and an abundant supply of pleasant information, he beguiled the hours of suffering, and rendered his presence acceptable as a consoling friend, as well as an efficient physician. He was, therefore, greatly beloved by those who depended on him for relief. Following him, as I have done, in his practice in this city, it has been a gratifying circumstance to listen to the praises of one who, while he occupied the elevated position which we have described, would bend to the voice of suffering humanity, and pour consolation and peace into the hearts of those who were looking to him for deliverance from corporeal sufferings. He was an amiable man, although endowed with strong feelings and a quick disposition, which would occasionally be manifested, like the lightning's flash, he never bore enmity, and soon returned to the enjoyment of the kindlier feelings of his nature."

